# The role of the active site tyrosine in the mechanism of lytic polysaccharide monooxygenase†

**DOI:** 10.1039/d0sc05262k

**Published:** 2020-11-04

**Authors:** Aina McEvoy, Joel Creutzberg, Raushan K. Singh, Morten J. Bjerrum, Erik D. Hedegård

**Affiliations:** Division of Theoretical Chemistry, Lund University Box 124 SE-221 00 Lund Sweden erik.hedegard@teokem.lu.se; Department of Chemistry, University of Copenhagen Copenhagen Denmark

## Abstract

Catalytic breakdown of polysaccharides can be achieved more efficiently by means of the enzymes lytic polysaccharide monooxygenases (LPMOs). However, the LPMO mechanism has remained controversial, preventing full exploitation of their potential. One of the controversies has centered around an active site tyrosine, present in most LPMO classes. Recent investigations have for the first time obtained direct (spectroscopic) evidence for the possibility of chemical modification of this tyrosine. However, the spectroscopic features obtained in the different investigations are remarkably different, with absorption maximum at 420 and 490 nm, respectively. In this paper we use density functional theory (DFT) in a QM/MM formulation to reconcile these (apparently) conflicting results. By modeling the spectroscopy as well as the underlying reaction mechanism we can show how formation of two isomers (both involving deprotonation of tyrosine) explains the difference in the observed spectroscopic features. Both isomers have a [TyrO–Cu–OH]^+^ moiety with the OH in either the *cis*- or *trans*-position to a deprotonated tyrosine. Although the *cis*-[TyrO–Cu–OH]^+^ moiety is well positioned for oxidation of the substrate, preliminary calculations with the substrate reveal that the reactivity is at best moderate, making a protective role of tyrosine more likely.

## Abstract 2

With QM/MM, we investigate the mechanism of tyrosine deprotonation in lytic polysaccharide monooxygenases. Our results support deprotonation and our calculated UV-vis spectra show that two isomers must be formed to match recent experiments.

## Graphical abstract

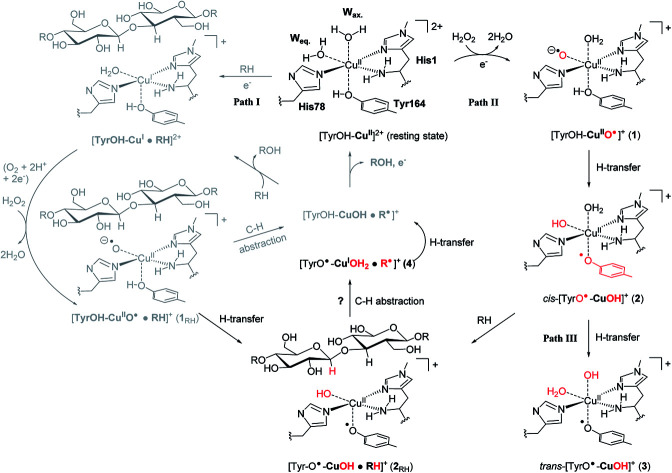

## Introduction

Carbohydrate polymers such as starch, cellulose, and chitin comprise a large, renewable resource, both as alternatives to fossil fuel and as a carbon source for commercial chemicals.^1^ Yet, exploitation of this resource requires energy-efficient polysaccharide degradation, which is currently limited by the inherent recalcitrance of many naturally occurring polysaccharides.

**Scheme 1:**
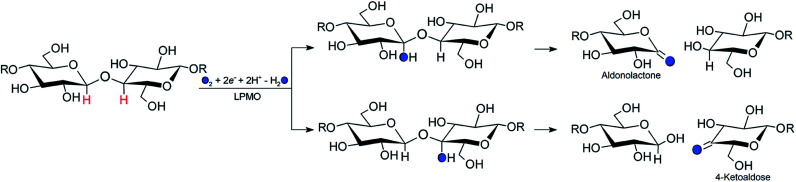
Regioselective oxidation of the glycoside bond in cellulose by the LPMO catalysed reaction and with a co-substrate (O_2_ or H_2_O_2_). Hydroxylation of the glycosidic bond occurs at either the C1 (top pathway) or the C4 position (bottom pathway). Filled circles and R represent oxygen atoms and glycosyl unit, respectively.

The enzymes that are now called lytic polysaccharide monooxygenases (LPMOs) have been discovered to boost^1–6^ polysaccharide degradation and have been developed into a key ingredient for efficient polysaccharide decomposition. Originally, this decomposition was believed to be solely hydrolytic until studies on the chitinolytic bacterium *Serratia marcescens*^3^ showed that LPMOs employ oxidative chemistry: the LPMOs catalyze oxidation of the otherwise unreactive glycosidic C–H bonds on either the C1 or C4 (or both) carbons in cellulose and other polysaccharides,^3,7^ ultimately disrupting the polysaccharide surface and thus boosting the degradation. The overall reaction catalyzed by LPMO is shown in Scheme 1.

**Fig. 1:**
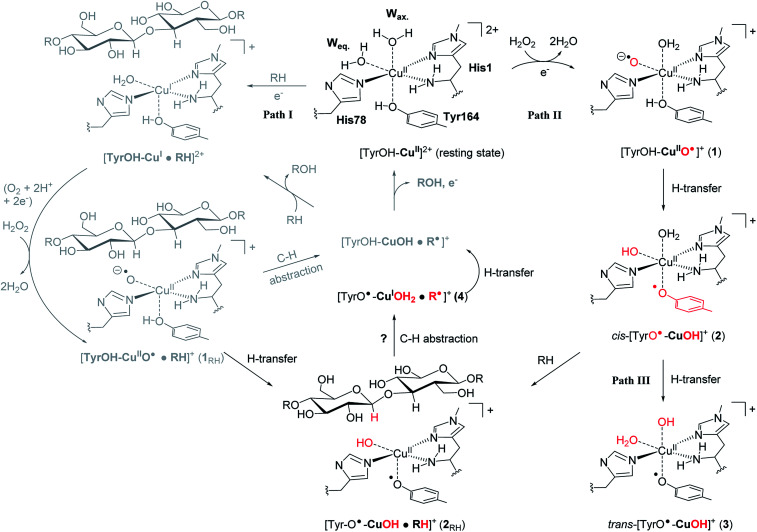
The LPMO active site in its resting state, [Tyr–OH–Cu]^2+^, showing the histidine brace (with labels from PDB 5ACF^26^). Reaction path I (gray) is the consensus mechanism upon substrate (RH) binding and with O_2_ or H_2_O_2_ as the co-substrate. Reaction paths II and III are investigated here as reactions with H_2_O_2_ before binding of the substrate, leading to intermediates with de-protonated tyrosine. Red-colored parts indicate atoms that have been chemically modified. The [CuOH]^+^ moiety has no formal oxidation states (see the text).

The first discovered members^2,3^ of the LPMO family are now denoted as “Auxiliary Activity (AA)” enzymes AA9 and AA10 and many additional members (from AA11 and AA13 to AA16) have been categorized since the original discovery.^8–14^ The different LPMOs have somewhat different amino-acid sequences (even within the same families), and target a wide range of different polysaccharide substrates.^15–18^ Despite this variation, a common feature is an active site with a copper ion, coordinated by two histidine residues which are known as the histidine brace^8^ (see Fig. 1).

The LPMOs are already employed to boost industrial polysaccharide degradation,^1,4,5,19,20^ but we are still far from reaching the full potential. A significant step in the direction of better utilization of LPMOs could be achieved by elucidation of the molecular mechanism. Despite recent focus from both experimental and theoretical researchers,^21–25^ crucial parts of the molecular mechanisms have remained controversial.

An (simplified) overview of the consensus mechanism is given on the left side (Path I) of Fig. 1, using a C4 oxidizing AA9 LPMO as an example. In Path I, the resting state is reduced, followed by substrate (RH) binding, although the specific order of reduction and substrate binding is not clear. The reduced state can react with an oxygen source to form a reactive intermediate, which oxidizes the polysaccharide substrate. The source of oxygen (co-substrate) is one of the controversial topics, as both H_2_O_2_ and O_2_ have been suggested as natural co-substrates^27,28^ (both are included in Path I). Another controversial part is the identity of the reactive intermediate: early studies suggested a Cu(ii)-superoxide,^29–31^*i.e.*, a complex with a [CuO_2_]^+^ core, formed by reaction of the (reduced) resting state and O_2_. Theoretical and experimental studies have later shown that the superoxide species may be formed,^32–34^ but it is not a powerful enough oxidant to attack polysaccharide C–H bonds. Instead, it has been suggested^4,23,35^ that Cu(iii)-hydroxide ([CuOH]^2+^) or Cu(ii)-oxyl ([CuO]^+^) complexes^21–25^ are responsible for the C–H abstraction. Fig. 1 assumes an oxyl complex (1_RH_), which reacts with the substrate by abstracting a C4–H hydrogen atom to form [TyrOH–CuOH·R˙]^+^, followed by a recombination step. Yet another topic that has puzzled LPMO researchers is the role of the aromatic residues close to the active site: a tyrosine (Tyr164 in Fig. 1) is positioned with the OH group close to the axial coordination site of copper.^26^ This tyrosine is found in all LPMO classes but is absent in most AA10 LPMOs (where it is replaced by phenylalanine).^36^ Replacing tyrosine with phenylalanine in an AA9 LPMO has been found to reduce the activity significantly,^1,2,36^ which suggests that the tyrosine is important. One possibility is that the LPMOs' catalytic machinery with the tyrosine and that without the tyrosine differ, *e.g.*, the tyrosine could stabilize certain intermediates involved in substrate oxidation.^20^ This is supported by experimental observations showing that photo-reduction more readily takes place in AA10 LPMOs lacking tyrosine as well as differences in their EPR-spectra, suggesting different electronic structures.^9,37^ An alternative proposal is that the tyrosine is part of an electron transfer chain from the reducing agent to the copper.^31^

The role of tyrosine can also be to protect the active site against harmful oxidation in the absence of the substrate, as suggested by two recent studies. The two studies have (in the absence of the substrate) managed to detect and spectroscopically characterize a radical intermediate, involving a deprotonated tyrosine.^38,39^ Both studies obtained well-defined optical spectra by adding H_2_O_2_ and an electron donor (ascorbate) to an AA9 LPMO. Intriguingly, one of the studies obtained an inactive intermediate, formed irreversibly,^39^ whereas the other^38^ found that the intermediate was obtained in a reversible process that did not prohibit oxidation of the substrate (and hence, the intermediate could either be directly or indirectly involved). The two studies also proposed different spin-states and obtained remarkably different optical spectra.^38,39^ These results were somewhat surprising and may seem contradictory, since the two studies employed LPMOs from the same family (AA9). Unfortunately, no explanation for the above differences could be given in ref. 38 and 39 and no mechanism for the formation of the intermediates was proposed (one of the studies^39^ did, however, suggest a structure for their proposed intermediate based on DFT calculations). Notably, experimental conditions in the two studies were vastly different: although the same LPMO family was used, the two studies employed different family members, namely TaLPMO9A^38^ and LsAA9,^39^ respectively. For the former, the reaction with H_2_O_2_ was carried out in slightly acidic pH and sub-equimolar concentrations of H_2_O_2_ while for the latter, maximum conversion was achieved at pH 10 with excess H_2_O_2_.

Using an approach based on density functional theory (DFT), combined with molecular mechanics (QM/MM), we here propose that the spectroscopic differences indeed have origin in different species and we suggest a mechanism for their formation: our mechanism follows Path II in Fig. 1: since previous theoretical studies including the substrate^23–25^ (Path I) have shown that oxyl species can be formed from the reductant and H_2_O_2_, Path II departs from [Tyr–OH–CuO]^+^ (1), abstracting H from tyrosine. This reaction (1 → 2) forms a *cis*-[TyrO–CuOH]^+^ moiety (2) with a deprotonation of tyrosine. We propose that 2 is the intermediate observed in ref. 38. We next propose that *trans*-[TyrO–CuOH]^+^ (3) can be formed by internal hydrogen transfer (Path III in Fig. 1). Notably, our proposed structure of 3 is similar to the intermediate proposed in ref. 39 The nature of 2 and 3 is confirmed by calculations of UV-vis spectra with our QM/MM optimized structures, reproducing the main features of the measured spectra with high accuracy. Since most other theoretical studies on the mechanism^21–24^ have not considered deprotonated tyrosine, we have done a preliminary test of whether intermediate 2 is capable of abstracting hydrogen from a glycosidic C–H bond, thus joining Paths I and II as shown in Fig. 1. This is done by introducing the substrate at the stage of 2 (we denote this as 2_RH_ in Fig. 1) and calculate the corresponding activation and reaction energies for the C–H abstraction.

## Computational details

### General

All structure optimizations and energy calculations on the mechanism were performed with a QM/MM approach, using the QM software Turbomole (versions 7.1 and 7.2)^40^ and the MM software AMBER 14.^41^ The QM/MM calculations were performed with the ComQum interface,^42,43^ which combines these two programs in an electrostatic embedded QM/MM formalism. The QM/MM calculations were generally carried out in the same manner as our previous calculations. We therefore only provide a brief overview of the calculations while we refer to previous investigations^23,34^ as well as the ESI† for further details (*e.g.* protein setup, protonation states and initial equilibration). All QM/MM calculations employed density functional theory (DFT) for the QM region (the sizes of QM regions are specified in the ESI†). The structure optimizations are carried out with the dispersion-corrected TPSS-D3 functional,^44,45^ in combination with a def2-SV(P) basis set (unless otherwise specified).^46,47^ Calculations with the TPSS functional always employed the resolution of identity (RI) approximation with standard auxiliary basis sets (as implemented in Turbomole). The final energies are obtained as single-point calculations, employing a def2-TZVPP basis set (with an auxiliary basis set of the same size).^46^ For spin-state and reaction energetics, additional calculations were done (as single-point calculations) with the B3LYP-D3 ^45,48–50^ functional and def2-TZVPP basis set on structures optimized as described above. Although for brevity we denote the functionals TPSS and B3LYP in the paper, all calculations of structures and energies include dispersion corrections. The reaction and activation energies are calculated as linear transit calculations without thermochemical or zero-point vibrational energy corrections. Such corrections have previously been shown to be small for H-atom abstractions.^51–53^ We locate the TS as the highest energy structure in the linear transit calculations, *cf.* Fig. S3–S8 in the ESI.†

### UV-vis spectra

UV-vis spectra were calculated for intermediates 2 and 3 in both *S* = 1 and *S* = 0 spin states. For the latter, we employed an open-shell formulation for 2 and a closed-shell formulation for 3. We additionally carried out calculations of the resting state (*S* = 1/2) and two models of 2 and 3 without axial and equatorial water ligands, respectively. The TD-DFT calculations included 45 states (roots).

All TD-DFT calculations were done with Gaussian^54^ employing the CAM-B3LYP^55^ functional in combination with the def2-TZVPP basis set (both employed as implemented in the Gaussian program). The calculated energies and oscillator strengths were convoluted with a Gaussian function (with a broadening factor of 0.3 eV).

## Results and discussion

### Spin-states of 1, 2 and 3

The three intermediates 1–3 in Fig. 1 can attain both singlet and triplet spin states. Since this was a point of disagreement between the experiments suggesting deprotonation of tyrosine,^38,39^ we initially probe the energy-difference between the singlet and triplet spin states. The obtained splittings are given in Table 1. For 1, the triplet and singlet states have previously been calculated to be close in energy, with the triplet as the ground state.^19,21,29,45^ In this case, the two spin states can be interpreted as a result of the spin from the unpaired electron on the d^9^ Cu(ii) being either ferromagnetically coupled (triplet) or antiferromagnetically coupled (singlet) to the unpaired electron on oxyl (O˙^−^), *i.e.* the singlet is an open-shell singlet. As expected, the triplet and open-shell singlet for 1 are indeed close in energy. The triplet state is the most stable and we obtain a splitting between 5 and 15 kJ mol^−1^ (depending on the functional).

**Table 1:** Spin state splittings (Δ*E*_QMMM_ = *E*_Singlet_ − *E*_Triplet_ in kJ mol^−1^). The values are, unless otherwise noted, obtained from def2-TZVPP single-point calculations on structures obtained with TPSS-D3/def2-SV(P)

Intermediate	TPSS-D3	B3LYP-D3
1	4.5	15.7
2 a	8.0^a^	10.9^a^
2 (closed-shell singlet)	21.0	63.8
2 (def2-TZVPP structure)^b^	5.1	3.8
3 (closed-shell singlet)^c^	−20.3	−10.6
1_RH_	6.9	16.5
2_RH_	4.2	0.4

a On the level employed for structure optimizations, the calculations always converged to a closed-shell singlet, also when the calculations were started from a density obtained from a triplet.

b Here the QM/MM structure optimization was performed with def2-TZVPP, yielding an open-shell singlet.

c The open-shell singlet is close to identical, yielding splittings of −19.0 (TPSS-D3) and −15.8 (B3LYP-D3) kJ mol^−1^, respectively.

The spin-state splitting of 2 shows that the (open-shell) singlet and triplet also are rather close in energy for this intermediate: the triplet is most stable with both functionals, but only with 8 kJ mol^−1^ (TPSS) or 11 kJ mol^−1^ (B3LYP). The closed-shell singlet was always found to be higher in energy for both TPSS and B3LYP, although for the latter functional, this was most pronounced (see Table 1). For 2 we also probed the effect of increasing the basis set size to def2-TZVPP during structure optimization, but this change has only a small effect on the energetics and the structures.

It should be mentioned that DFT occasionally struggles with spin-state splittings: recent highly accurate calculations on LPMO intermediates with multiconfigurational wave functions (CASPT2) have shown that spin-state splittings can be somewhat functional dependent, and only qualitative results can be expected for oxygen-bound LPMO intermediates.^56^ All conclusions in the paper will therefore be based on results from two different DFT functionals to ensure that potential mismatches between the functionals are accounted for in our conclusions. Furthermore, both triplet and (closed and open-shell) spin-states have been investigated in the reactions below. With these precautions in mind we also comment on spin-states in the experimentally observed intermediate described in ref. 38 (which we here propose is 2). Based on X-band EPR spectroscopy, it was suggested that a singlet state was present at low temperature (77 K) but with a populated triplet at room temperature, *i.e.*, the energy difference between the two states is indeed small, suggesting that both triplet and singlet states are present in solution. Within the expected accuracy of DFT, our results fit well with this conclusion.

For 3, we find that the singlet is the most stable (the triplet is between 10 and 20 kJ mol^−1^ higher, depending on the employed functional). The values reported in Table 1 are for the closed-shell singlet as the open-shell singlet gave rise to almost identical energetics (and structures). A singlet ground-state is consistent with the observations in Paradisi *et al.*,^39^ although the QM-cluster DFT calculations by Paradisi *et al.* employed an open-shell formalism for the singlet.

The calculation of spin-state energetics also allows a few comments on the electronic structures of 1–3. The electronic structures of 1 and 2 are best discussed in terms of their spin density (see Table 2). To relate the spin-density to a Cu oxidation state it can be noted that Cu(ii) and Cu(iii) are d^9^ and d^8^ systems, respectively. The d^9^ formulation will generally carry spin-density, while d^8^ can be either a closed-shell singlet (*i.e.* with zero spin density) or a triplet (*i.e.* with non-zero spin-density). Thus, it may be difficult to give an unequivocal assignment of the formal oxidation state, but the spin-density on Cu together with the spin-density on the remaining ligands can give an indication. For 1 both O_ox_ and Cu in the [CuO]^+^ core carry significant spin-density, which fits well with a Cu(ii) ion and oxyl(O˙^−^) radical, here spin-coupled to a triplet. It can also be noted that there is a small amount of spin density delocalized to the terminal (coordinating) amino group of His1 (N_ter_).

**Table 2:** Mulliken spin densities for selected intermediates, 1, 2 as well as 1_RH_ and 2_RH_ (all for *S* = 1). Labelling on tyrosine follows usual PDB labels. All values are from TPSS-D3/def2-TZVPP

Complex	[CuO(H)]^+^		N_ter_	Tyrosine						
Atom	Cu	O_ox/hyd_	N_ter_	O_Tyr_	C^ζ^	C^ε1^	C^δ1^	C^γ^	C^δ2^	C^ε2^
1	0.56	1.15	0.08	0.00	0.00	0.00	0.00	0.00	0.00	0.00
2	0.69	0.25	0.11	0.31	−0.02	0.12	−0.08	0.24	−0.07	0.16
1_RH_	0.48	1.21	0.08	0.00	0.00	0.00	0.00	0.00	0.00	0.00
2_RH_	0.61	0.30	0.13	0.31	−0.01	0.17	−0.08	0.25	−0.07	0.18

For 2, the spin-density on Cu increases slightly, while it decreases significantly on oxygen in the OH group in [CuOH]^+^ bound to Cu. Furthermore, significant spin density is introduced on the deprotonated tyrosine ligand. Thus, an interpretation with Cu(iii) bound to OH^−^ and a negatively charged tyrosine does not seem to fit these spin-densities (as this would lead to spin-densities close to zero on the tyrosine and OH ligands). The large spin-density on tyrosine rather suggests a tyrosine radical coupled to a [CuOH]^+^ unit, where the spin is delocalized over all atoms in the latter, leaving assignment to one specific oxidation state of the copper atom difficult. Hence, no formal oxidation states are given for the [CuOH]^+^ moiety in Fig. 1.

For 3, the closed shell singlet spin-state indicates a *trans*-[CuOH]^+^ moiety comprised of a Cu(iii) d^8^ ion (in a closed-shell configuration), bound to a hydroxide (OH^−^) and a deprotonated Tyr–O^−^.

Finally, we have probed if substrate binding would perturb the active site sufficiently to change the spin-state splitting for 1_RH_ and 2_RH_. However, as seen from Table 1, the spin-state splitting is almost identical to 1 and 2 (and likewise for their electronic structures in Table 2).

### UV-vis spectra

**Fig. 2:**
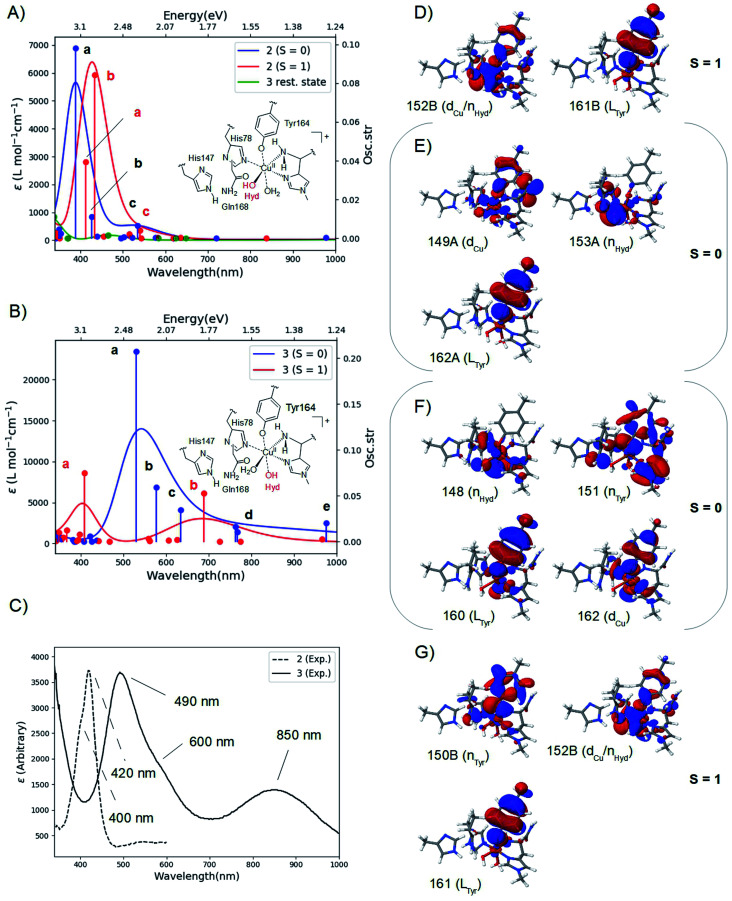
Calculated spectra for the resting state, 2 (A) and 3 (B) with CAM-B3LYP/def2-TZVPP from TPSS/def2-SV(P) QM/MM structures. The experimental spectra^38,39^ are shown in (C), with the intensity of 2 scaled. Intense transitions are labelled a–e. Selected, involved orbitals are shown for 2 in (D) and (E) and for 3 in (F) and (G). The orbitals are labeled after their main character and by their numbers (see the ESI† for further details).

The calculated spectra are shown in Fig. 2A for 2 and Fig. 2B for 3 while the two experimental spectra are shown in Fig. 2C (calculated transition energies and oscillator strengths are compiled in Tables S11–S14†). We include both *S* = 1 and *S* = 0 states (shown in red and blue, respectively). For 2, the most intense peaks are (for both spin states) comprised of two transitions (labelled a and b in Fig. 2A). For the triplet, transition a is at 412 nm (3.00 eV) and transition b is at 433 nm (2.85 eV). These transitions are in excellent correspondence with the experimental room temperature absorption spectrum, whose most intense peak is around 420 nm (2.95 eV). The singlet a and b transitions are slightly higher in energy at 388 nm (3.20 eV) and 426 nm (2.91 eV), but still in good correspondence with the experimental spectrum (see further the Discussion section). For comparison, we also calculated the spectrum of the resting state (green curve in Fig. 2A). As expected, this state shows no intensity in this energy interval.

We next analyzed the transitions a and b in terms of major orbital contributions. Selected orbitals are shown in Fig. 2D and E (see further Tables S11 and S12†). The most intense transition (a for the singlet, b for the triplet) are metal-to-ligand charge transfer (MLCT) excitations, involving transitions from orbitals of Cu d-character (d_Cu_ in Fig. 2E), orbitals with hydroxyl lone-pair character (n_Hyd_ in Fig. 2E) or a mixture of these two (d_Cu_/n_Hyd_ in Fig. 2D). All transitions are exclusively to an orbital of π-character on tyrosine (L_tyr_). The large involvement of the tyrosine orbitals explains how deprotonation of tyrosine can lead to the experimentally observed changes in the absorption spectrum. The two spectra additionally involve several transitions (collectively denoted as c in Fig. 2A and B) of lower intensity. These transitions involve mainly second coordination sphere ligands (Gln162) and tyrosine (*cf.* Tables S11 and S12†). Moving to 3 in Fig. 2B, our calculations predict that the most intense peak around 500 nm is due to the singlet. This peak is comprised of three transitions (labelled a–c in Fig. 2B and Table S13†). Using the most intense transition (a) at 530 nm (2.34 eV) as the outset, this transition is in good correspondence with the most intense peak in the experimental spectrum, observed at 490 nm (2.53 eV). Transition a is a ligand-to-metal charge transfer (LMCT) excitation, involving transitions from ligand orbitals on the hydroxide and tyrosine (n_Hyd_ and L_Tyr_ in Fig. 2F and Table S13†) to an orbital of d-character (d_Cu_). The transitions labelled b and c are also of LMCT character, in the former case from orbitals on the non-coordinating histidine (L_His147_ Table S13†) and in the latter case from n_Hyd_. Finally, the calculated spectrum in Fig. 2B contains transitions of LMCT character with lower intensity around 762–767 nm (1.62–1.63 eV) and 976 nm (1.27 eV); these transitions involve tyrosine and histidine orbitals (His1 and His147) and are marked with d and e in Fig. 2B (and Table S13†). They are in reasonable correspondence with the experimentally observed (rather broad) transitions with maximum at 850 nm (1.46 eV). Notably, our calculated UV-vis spectrum for 2 shows no transitions in this region. Fig. 2B also shows the calculated spectrum from the triplet spin-state, and orbitals involved in the most intense transitions (labelled a) are shown in Fig. 2G. The transitions for the triplet are expected to be masked by the more intense transitions from the singlet.

We have additionally investigated two five coordinated species (obtained by removing either the weakly coordinating W_ax._ or W_eq._); we return to these calculations in the Discussion section.

### Reaction mechanism

**Fig. 3:**
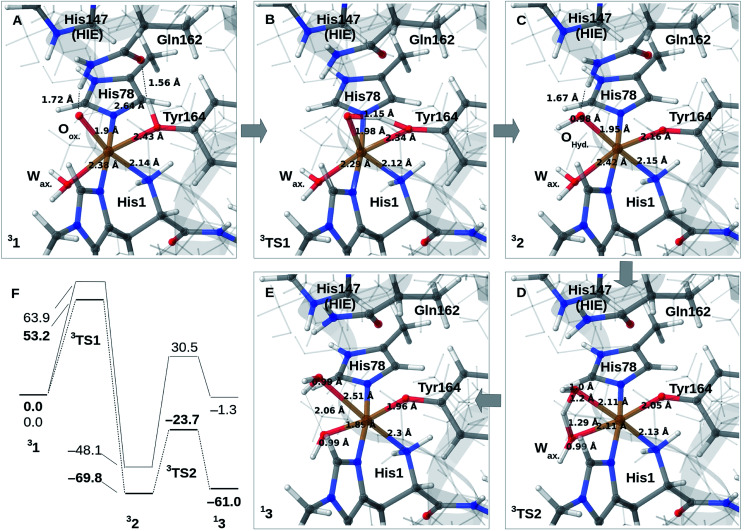
(A)–(C) shows the reaction mechanism for 1 → 2 (Path II) where (A) is 1, (B) is the transition state and (C) is 2. From (C), reaction 2 → 3 (Path III) is shown in (D) and (E) where (D) is the transition state and (E) is 3. (F) is the energy diagram (in kJ mol^−1^ with 1 as the reference). Only the most stable spin-states are shown (the multiplicities are indicated in superscript). All structures are obtained with TPSS-D3/def2-SV(P), while the energies in (F) are obtained with def2-TZVPP, employing TPSS (bold) or B3LYP.

We finally investigate reaction and activation energies from the formation of 2 and 3 through H-transfer reactions (Paths II and III in Fig. 1). An energy diagram, the QM/MM optimized structures of 1–3, as well as transition states, connecting 1 and 2 (TS1) and 2 and 3 (TS2) are shown in Fig. 3A–F. Before discussion of the individual reaction, we note that all reactions show large (mainly electrostatic) contributions from the protein environment (*cf.* Tables S1–S3†), underlining the importance of the QM/MM treatment. In the first reaction (1 → 2), hydrogen is transferred from the tyrosine hydroxy group to the oxyl (O_ox._ in Fig. 3A) on the copper, forming a *cis*-[CuOH]^+^ moiety. In the next reaction (2 → 3), an H-atom is transferred from the axial water molecule (W_ax._ in Fig. 3A–C) to the *cis*-[CuOH]^+^ moiety formed in the first reaction.

For reaction 1 → 2, both functionals are in reasonable agreement and predict the overall reaction as thermodynamically favorable and with activation energies suggesting a kinetically feasible reaction (see Fig. 3F): the reaction energy is −70 kJ mol^−1^ (−48 kJ mol^−1^ with B3LYP) while the activation energy is 53 kJ mol^−1^ (64 kJ mol^−1^ with B3LYP). As seen from Fig. 3F, we report the activation energy from the triplet state of TS1, which is the most conservative value (see the ESI† for further discussion of this point). From the structures in Fig. 3A–C, we see that during the reaction, the Cu–O_Tyr_ bond distance changes significantly from 2.43 (1) to 2.16 Å (2), *i.e.* the tyrosine coordinates after deprotonation. This explains the large spectral changes from 2 with intense MLCT transitions, compared to the resting state (where the QM/MM optimized Cu–O_Tyr_ distance is 2.43 Å). The Cu–O_ox_ bond length only shows a minor change indicating that this bond is already long in 1 in accordance with the high reactivity of the oxyl species. Note also that W_ax._ in 2 is rather weakly coordinated with a Cu–O distance of 2.42 Å (Fig. 3C).

The next part of the reaction (2 → 3) involves (for both functionals) a transition from the triplet to the singlet potential energy surface. Therefore, it is not surprising that DFT shows a more pronounced functional dependency: the reaction energy is 9 kJ mol^−1^ with TPSS, and somewhat more uphill with B3LYP (49 kJ mol^−1^). For both functionals, the overall reaction (1 → 3) is either downhill with −33 kJ mol^−1^ (TPSS) or roughly neutral with −1 kJ mol^−1^ (B3LYP). The functional dependence is also seen for the activation energy, where TPSS and B3LYP obtain 46 and 79 kJ mol^−1^, respectively (*cf.*Fig. 3F and Table S6†). Thus, while the TPSS results suggest a reaction that is certainly feasible, the B3LYP reaction- and activation energies are less favorable. Yet, both the activation and reaction energy from B3LYP are not large enough to rule out reaction 2 → 3, and we therefore keep this path as a possibility, although more accurate methods are clearly desirable. We are currently working with obtaining more accurate (CASPT2) reference values for reaction 2 → 3. In the case of the activation energies for reaction 2 → 3, we have used the triplet state for TS2 as seen from Fig. 3F. Yet, the singlet gives almost identical energies (and structures, see further the discussion in the ESI†). In addition, it should be noted that the change of spin-state during the reaction means that spin–orbit coupling will likely influence the kinetics.

The formation of 3 leads to a relatively long Cu–O bond (2.51 Å) for the formed equatorial water molecule (Fig. 3E), while the formation of the hydroxy (OH^−^) from the axial water (W_ax._) leads to shortening of this Cu–O bond (from 2.42 Å in Fig. 3C to 1.89 Å in Fig. 3F). Most important for the spectroscopy is perhaps that the Cu–O_Tyr_ distance remains short (1.96 Å) in 3.

**Fig. 4:**
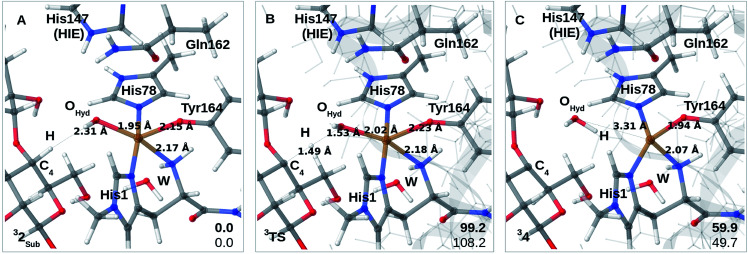
(A)–(C) is the reaction mechanism for the reaction 2_RH_ → 4 where (A) is 2_RH_, (B) is the transition state and (C) is 4 (the multiplicities are indicated in superscript). Energies are given in kJ mol^−1^ (lower right corner; bold is TPSS values and the other value is B3LYP).

Finally, we have also probed how well the *cis*-[CuOH]^+^ moiety in 2 reacts with the substrate. Note that we do not consider 3 in the presence of the substrate since substrate binding likely leads to dissociation of W_ax._,^26^ making 3 unlikely to form in the presence of the substrate. We added the substrate to 1 and 2 (see the ESI† for details) to first investigate if the substrate significantly changes the relative energies of 1 and 2, but this turned out not to be the case: the reaction energy for 1_RH_ → 2_RH_ is −73 kJ mol^−1^ with TPSS and −52 kJ mol^−1^ with B3LYP (*cf.* Table S10†), and thus quite similar to reaction 1 → 2. We next investigated whether Path II could be joined with Path I, *i.e.*, whether 2_RH_ is involved in C–H abstraction from C4 (2_RH_ → 4 in Fig. 1). The results are shown in Fig. 4A–C. The activation energies are on the higher side, between 99 and 108 kJ mol^−1^, and the reaction is somewhat uphill with 60 or 50 kJ mol^−1^ (depending on the functional), making the reaction less likely.

## Discussion

The calculated spin-states and spectra reveal (within the limits of DFT) that the intermediates observed in ref. 38 and 39 correspond to 2 and 3, respectively: experimentally, the most intense peak of intermediate 3 is red shifted 0.42 eV, compared to the most intense peak of 2 (2.95 eV in 2, compared to 2.53 eV in 3); our calculations predict a shift of 0.51 eV, using the most intense transitions b for 2 (*S* = 1) and a for 3 (*S* = 0), respectively (*cf.*Fig. 2A and B). We also note that since the spin-state splittings for both 2 and 3 are small, it is not unlikely that both triplet and singlet states are populated at room-temperature. We have therefore investigated spectra of both spin-states. In the case of 2, the calculated spectra of both singlet and triplet give rise to UV-vis spectra that correspond well to the observed UV-vis spectra. In fact, the most intense peak in the experimental spectrum (at 420 nm) has a shoulder around 400 nm (3.1 eV; *cf.*Fig. 2C). From our calculations a possible interpretation of this shoulder is that it corresponds to the singlet state, *e.g.*, transition a at 388 nm (3.20 eV). Alternatively, the triplet also shows a second intense transition (labelled a at 3.00 eV) which could give rise to the observed shoulder. Likewise, for 3 the most intense transition has a shoulder around 600 nm (2.1 eV; *cf.*Fig. 2C) and the calculated spectrum for the singlet shows several transitions that could give rise to this shoulder (*cf.* transitions b and c at 2.15 and 1.95 eV, respectively, in Fig. 2B). These transitions could explain the broader features of the intense peak in 3, compared to 2. Note that the intensity of the experimental spectrum of 2 is scaled to facilitate comparison with 3. The actual intensities are inaccurate since the exact concentrations of the observed intermediates are difficult to estimate. Thus, our TD-DFT calculations explain the different spectra observed in ref. 38 and 39 namely that it is due to the formation of 2 and 3, and their molecular and electronic structures corroborate well with available experimental evidence. The intermediate suggested by Paradisi *et al.*^39^ is rather similar to our 3, although without an equatorial water molecule (W_eq._). However, as described in the Results section, both W_ax._ in 2 and W_eq._ in 3 coordinated rather weakly, and their influence on the UV-vis spectra is expected to be small (we included the water molecules since the active site is solvent exposed). Calculations of spectra for 2 and 3 without water molecules indeed show that the effect is rather small (*cf.* Fig. S9 and S10†). The largest influence is found for 3 at longer wavelengths, where the experimental spectrum has a broad peak at 850 nm. In this region, the calculated spectra with and without W_eq._ (Fig. S10†) show peaks at each side of 850 nm. Thus, the broad features may be caused by the sensitivity of this peak to the solvent water, dynamically coordinating and de-coordinating.

We will now discuss possible roles of the active site tyrosine. As outlined in the Introduction, the intermediate detected by Paradisi *et al.*^39^ (our 3) is found to be inactive in substrate oxidation. It was instead suggested to protect the active site against oxidative damage in an uncoupled turnover (by a hole-transfer mechanism). Singh *et al.*^38^ also suggested a protective mechanism, but they also found that the conditions in which their intermediate (our 2) forms do not prevent LPMO oxidation of phosphoric acid swollen cellulose (PASC). Thus, a role in substrate oxidation cannot be excluded (in addition Singh *et al.*^38^ detected the formation of dityrosine formed after 2, which could be formed from 2 by a hole-transfer mechanism similar to what was suggested by Paradisi *et al.*^39^). Our suggested structures for 2 allow a closer investigation of its ability to abstract hydrogen from a polysaccharide substrate. The calculations with the substrate included (2_RH_) show that the activation and reaction energies of C–H abstraction are disfavored, compared to the consensus mechanism (Path I in Fig. 1): previous estimates of the activation energy in Path I with an oxyl intermediate (using the same LPMO) have been as low as 69–73 kJ mol^−1^ if His147 is in the HID form, while it is 104–111 kJ mol^−1^ with His147 in the HIE form.^23^ The reaction energies are, however, always negative for the oxyl intermediate, regardless of the His tautomer. With this large dependence on the second coordination sphere – and seeing that our calculations here employed the HIE form of His147 – we must be careful completely excluding 2_RH_ as an on-pathway intermediate. Yet, present results indicate that 2_RH_ at best is a secondary intermediate and that Path II in Fig. 2 most likely is off-pathway. Hence, it is more likely that tyrosine has a protective role, and the oxidative chemistry observed by Singh *et al.*^38^ was due to another (oxyl or yet unknown) intermediate. Notably, a protective role for tyrosine was very recently proposed for (yet another) LPMO from *Hypocrea jecorina*, by the group of Solomon:^57^ they reacted LPMO–Cu(i) with H_2_O_2_ which confirmed the generation of a Cu-bound tyrosine radical. No direct structural data for this intermediate were obtained, but the obtained absorption spectrum was very similar to the one reported by Singh *et al.*^38^ Solomon and co-workers proposed an anti-ferromagnetically coupled singlet for this intermediate. This is not unlikely seeing that our results show that triplet and singlet are close in energy and give rise to similar UV-vis spectra. While these results confirm that tyrosine radicals can be formed for different LPMOs, further studies should be directed at comparing different LPMOs to ensure that similar mechanisms of tyrosine deprotonation are possible. Focusing for the moment on TaLPMOAA9 and LsAA9, several similarities suggest that they can employ the same (or closely related) mechanisms: both Gln162 and His147 residues are present in TaLPMOAA9 (Gln173 and His164)^8^ and we find that both residues are involved in the mechanism. In 1, both His147 and Gln162 form H-bonds to the oxyl group and the Gln162 carboxyl group also forms a H-bond to the Tyr OH group (the two latter bonds are shown in Fig. 2A). The H-bond between carboxyl and the Tyr OH group is broken to form 2. The Gln162 residue also stabilizes 2 by a H-bond to the Cu-bound OH group (see Fig. 2C). Despite these similarities, there are also differences that could influence the mechanism: based on previous QM/MM optimizations^23,34^ and crystal structures^8,26^ the Cu–O_Tyr_ distance is between 0.1 and 0.6 Å longer in TaLPMOAA9. This difference could (in addition to the harsher experimental conditions in ref. 39) explain why 3 is not observed in ref. 38 and why 2 is not observed in ref. 39. When we chose here to use LsAA9 it was because several other theoretical investigations of C–H abstraction exist for this LPMO^22–24^ (usually involving oxyl intermediates). This allows direct comparison of 2_RH_ with previous theoretical estimates. When comparing different LPMOs, it will also be of interest to study whether formation of 2 and 3 could alternatively proceed through a hydroxyl radical. An OH-radical has been suggested to be part of the C–H activation mechanism (and was also proposed by Solomon and co-workers^57^): QM/MM studies on LsAA9 (ref. 23 and 24) suggested a rather structured (denoted as “caged” in ref. 24) OH radical with H_2_O_2_ coordinated to Cu and with an elongated O–O bond. However, both studies found this intermediate to be easily converted to an oxyl^23,24^ or hydroxyl/hydroxide^23^ intermediate. The same conclusion was recently reached by Bissaro *et al.*^25^ from QM/MM studies on an AA10 LPMO, suggesting (as ref. 23 and 24) an oxyl to be the active species in C–H activation.

## Conclusion

One of the open questions regarding LPMOs' boost of polysaccharide depolymerization has been the role of an active site tyrosine, strictly conserved in most LPMO families. Spectroscopic studies of two AA9 LPMOs have recently shown that chemical modification (deprotonation) can take place when LPMOs react with H_2_O_2_ without the substrate. However, the results were ambiguous since the two studies led to significantly different UV-vis spectra, suggesting that the underlying intermediates are different. The calculations presented here unite the two – apparently contradictory – experimental results: based on QM/MM calculations we show that two intermediates indeed can be formed; the intermediates are isomers and we suggest them to be 2 and 3 (see Fig. 1), respectively. We propose a mechanism for the formation of 2 from an oxyl complex (1 in Fig. 1) and next formation of 3 from 2 in an isomerization reaction. All reactions are energetically feasible, although we note that different DFT functionals do give rise to rather different energetics for reaction 2 → 3. We are currently investigating the reactions with methods based on multiconfigurational wave functions.

To show that 2 and 3 indeed give rise to different spectra, we additionally modelled their optical spectra, employing TD-DFT. The calculated spectra commensurate with the experiment, largely reproducing the observed spectral features. In particular, the large shifts between the most intense transitions in the two experimental spectra are nicely reproduced.

We additionally probed the ability of 2 to abstract a hydrogen from a glycosidic C–H bond. The *cis*-[CuOH]^+^ moiety in 2 is less reactive than oxyl complexes; although activation energies are only slightly higher, the total reaction energies are somewhat uphill. Thus, the most likely role for tyrosine is to protect the active site against oxidative damage in the absence of the substrate.

The present study brings us one step further in elucidating the role of tyrosine, but there remain open questions. For instance, here we exclusively focus on one LPMO (LsAA9), while the experimental studies were carried out on different LPMOs (LsAA9 and TaLPMOAA9). Our future studies will focus on investigating how large impact the use of different LPMOs has on the spectroscopy as well as the proposed mechanism.

## Conflicts of interest

There are no conflicts to declare.

## Supplementary Material

SC-012-D0SC05262K-s001
