# Supplementary material for: The role of the active site tyrosine in the mechanism of lytic polysaccharide monooxygenase
Source: Chem Sci. 2020 Nov 4;12(1):352–62. doi: 10.1039/d0sc05262k (PMC8178957; doi:10.1039/d0sc05262k)
Supplement: SC-012-D0SC05262K-s001 [file SC-012-D0SC05262K-s001.pdf]

Electronic Supplementary Material (ESI) for Chemical Science.
This journal is © The Royal Society of Chemistry 2020

Supporting information

The role of the active site tyrosine in the mechanism of lytic polysaccharide monooxygenase

Aina McEvoy,[†] Joel Creutzberg,[†] Raushan K. Singh[‡], Morten J. Bjerrum ,[‡] and Erik D. Hedegård[†,*]

[†]*Division of Theoretical Chemistry, Lund University, Box 124, SE-221 00 Lund, Sweden*

[‡]*Department of Chemistry, University of Copenhagen, Copenhagen, Denmark*

E-mail: erik.hedegard@teokem.lu.se

Computational details.

QM/MM calculations setup: All QM/MM calculations were carried out with outset in a previously optimized structure of the resting state (cf. Figure S1 in the SI of Ref. [1]) and thus based on PDB 5ACF[2], including a tri-glycose substrate. Parts of the calculations were done without substrate to mimic conditions in recent spectroscopic studies.[3,4] For these calculations, the substrate was removed and the protein re-equilibrated (using the same procedure as in Refs. [1] and [5]). The equilibration included construction of a new set of charges, and these were obtained from a restrained electrostatic potential (RESP) fit for the active site. This fit employed a model of the resting state, shown in Figure S1 (A). It should be noted that the resting state without substrate contains an axial water molecule (W_{ax}. in Figure S1 A), which is not present in the substrate–LPMO complex[2] as it presumably dissociates in the substrate binding process. The water molecule was therefore included by introducing a coordinating crystal water from an X-ray structure of *Ls*AA9 LPMO without substrate (W2002 from PDB 5ACG[2]), modifying the Cu–O distance to 2.35 Å. All hydrogen positions in the resulting model (Figure S1 A) was optimized with TPSS-D3/def2-SV(P). With this setup, structures with and without substrate were optimized with QM/MM using the division between QM and MM regions described below.

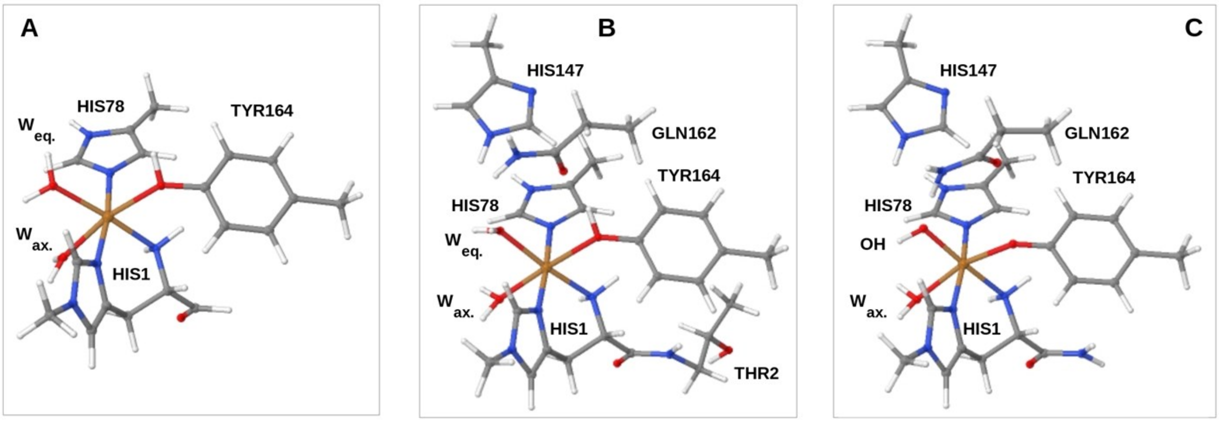

Figure S1: (A) Structure employed to obtain RESP charges (B) Example of QM region employed in this study (the resting state is used as example). (C) Truncated structure used for TD-DFT calculations. Labels refer to PDB 5ACF (and PDB 5ACG).

Spin-states and mechanism for formation of tyrosyl radical: The energetics of the spin-state splitting of **1**–**3** and reactions forming **2**, **3** as well as $\mathbf{1}_{RH}$, $\mathbf{2}_{RH}$ and **4** (see Figure 1 in the main paper) were investigated with QM/MM. An example of the employed QM system is shown in Figure S1 (B), using the resting state (without substrate) as example. For **1** and **2** the $W_{eq.}$ is (formally) replaced by $O^{\bullet -}$ and OH^{-}, respectively. In **3**, $W_{ax.}$ is replaced by OH^{-}. For the systems without

substrate, the QM systems, in addition to $H_2O/O^{\bullet -}/OH^-$ ligands and the copper ion, consisted of the remaining first coordination sphere i.e. His1, the imidazole ring of His78, and the phenol ring of Tyr164, the two latter capped with a hydrogen atom replacing C_α. The entire His1 residue (which coordinates to Cu through the terminal amino group) as well as the imidazole side chain, was included. Further, the neighboring Thr2 residue was included up to the C_α atom, which was replaced by a hydrogen atom. Finally, two second-sphere residues were included: His147 and Gln162 were included as the sidechain up to C_β as both side chains may be involved in hydrogen bonding to the oxygen atom in the $[CuO]^+$ or $[CuOH]^+$ moieties. His147 was included in the HIE form. Structures including the substrate additionally included the two glucose rings of the substrate in the QM region (the third was described by MM), in complete analogy to our previous study.[1] For this study, we additionally for substrate-bound intermediates decided to include the "pocket" water,[2] connecting the terminal NH_2 group to the substrate through hydrogen bonding. In all cases where the boundary between QM and MM regions comprise a covalent bond, the hydrogen link-atom approach is employed: the QM region is capped with hydrogen atoms, the positions of which are linearly related to those of the corresponding carbon atoms in the full system.[6,7] The MM part was kept fixed in all structure optimizations, since our previous studies have shown that with a large QM region (as used here) the effect of relaxing the MM region is small after Cu(I) has been oxidized.[1,5] The small contributions from ΔE_{MM} suggests that this is also the cases here (the ΔE_{MM} contribution is generally below ΔE_{ptch} , see Tables S1–S3 and Figure S3–S8 below).

The total QM/MM energy is calculated as

$$E_{QMMM} = E^{QM}_{ptch} + E^{total}_{MM} - E^{QM}_{MM} \tag{1}$$

where E^{QM}_{ptch} is the energy of the QM region calculated with DFT, including hydrogen link atoms and a point-charge model of the MM region (taken from the Amber force field and excluding only the carbon link atoms).[8] The term E^{total}_{MM} is the total MM energy of the full system with the charges of the QM system zeroed and E^{QM}_{MM} is the MM energy of the QM region, still with zeroed charges; it is included to avoid double-counting of the energy of the QM system. In the results section we discuss the electrostatic effect of the protein, which can be calculated from

$$E_{ptch} = E^{QM}_{ptch} - E^{QM}_{vac} \tag{2}$$

where E_{vac}^{QM} is taken from a calculation in vacuum with the QM/MM optimized structure. Calculations on both singlet ($S = 0$) and triplet ($S = 1$) spin states were carried out. The singlet states of the intermediates **1**, **2** and **3** (as well as $\mathbf{1}_{RH}$ and $\mathbf{2}_{RH}$) were calculated as both open- and closed shell species, employing spin-unrestricted (broken symmetry) and restricted formulations, respectively. In the results section of the main paper, it will always be specified which of the two formulations we report. Note that the spin-state splitting is reported as obtained, and no corrections have been applied. In the present study, applying correction schemes will not lead to change of any conclusion (see tables S4–S9 for electronic energies as well as the expectation values $\langle S^2 \rangle$ for triplet and open-shell energies). We have refrained from applying corrections as we have previously seen that corrected spin-state splitting are not necessarily closer to highly accurate (CASPT2) calculations.[9]

UV-vis spectra calculations: The models employed for TD-DFT were slightly truncated compared to the QM systems from QM/MM optimizations (removing Thr2 to the amide N, which was truncated with a hydrogen atom, see Figure S1 C).

Spin-state splittings and reactions.

The main paper reports the spin-state splitting for **1, 2** as well as $\mathbf{1}_{RH}$ and $\mathbf{2}_{RH}$ in Table 1. As indicated in this table, calculations for the singlet spin state of **2** with TPSS-D3/def2-SV(P) resulted in a closed shell singlet (as judged from the zero spin-density). The open-shell singlet could only be obtained (with TPSS) by enlarging the basis set (to def2-TZVPP). Since we employ QM/MM with quite large QM regions, extending the employed basis set for structure optimization beyond def2-SV(P) is computationally expensive. Nevertheless, we decided to investigate **2** in this manner. While subtle changes between the closed-shell and open-shell singlets are seen between def2-SV(P) and def2-TZVPP basis sets, the obtained splitting for the latter is 5 kJ/mol (TPSS) or 4 kJ/mol (B3LYP), i.e., differences to the calculations with underlying structures obtained with TPSS/def2-SV(P) are quite small. The change to employ def2-TZVPP for structure optimization also has negligible effect on structures as indicated in Figure S2 (D). Hence, all optimization of structures in the paper have been carried out with TPSS-D3/def2-SV(P).

The small gaps between singlet and triplet states in **1** and **2** prompted us to investigate all reactions without substrate in both triplet and the singlet states. Moreover, the above-mentioned differences between def2-SV(P) and def2-TZVPP for the singlet state of **2** led us to investigate **2** and **3** in both

open- and closed-shell variants. As seen from Figure S2, the change in spin state has only small influence on the structures: the structural differences between triplet and singlet are shown for reaction **1** → **2** in Figure S2 (A)–(C) and for reaction **2** → **3** in Figure S2 (C), (E) and (F).

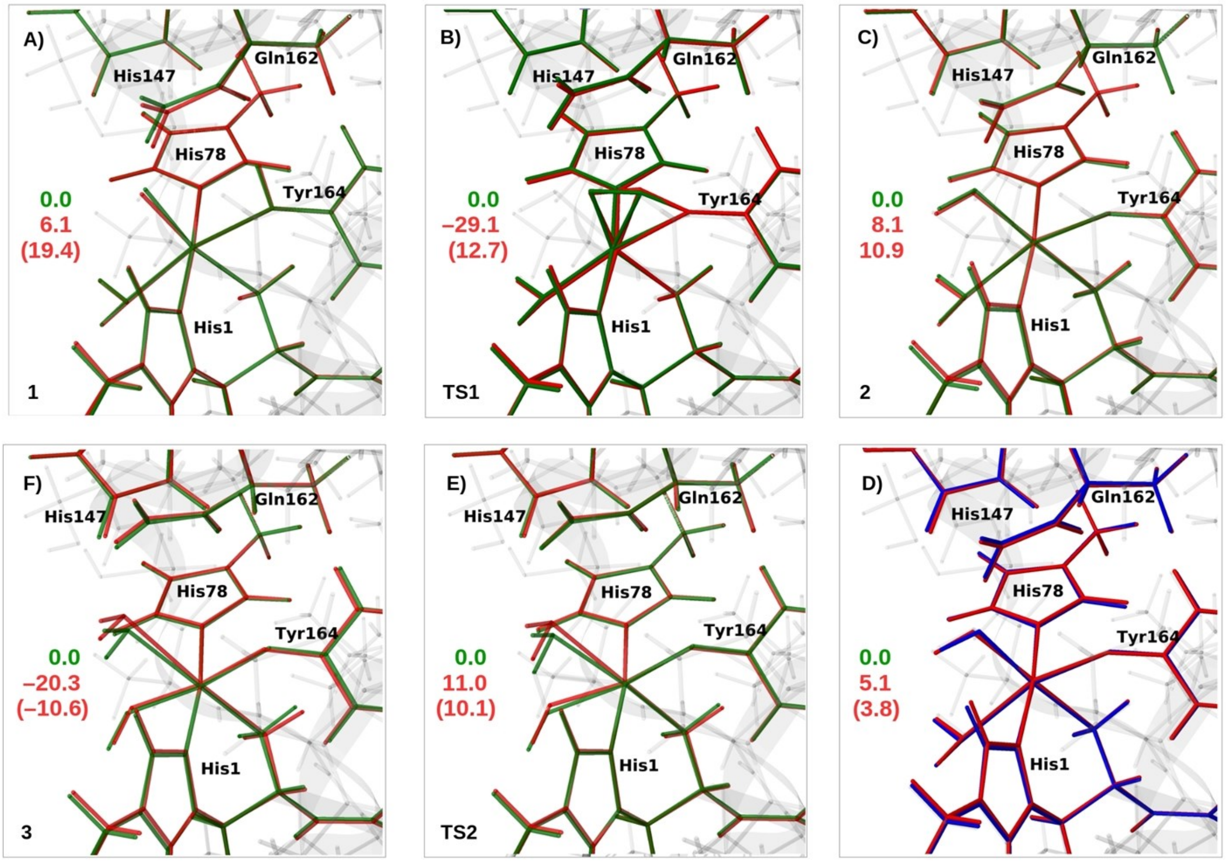

*Figure S2: Comparison of triplet (green) and singlet (red) structures for **1** (A), TS1 (B) **2** (C), TS2 (E) and **3** (F) obtained with TPSS/def2-SV(P). Figure (D) compares singlet structures of **2** obtained with TPSS/def2-SV(P) and def2-TZVPP (blue). The singlet-triplet splittings are also shown ($E_{Singlet}$– $E_{Triplet}$ in kJ/mol) and are always obtained as single point with TPSS and def2-TZVPP (B3LYP in parentheses).*

Still, the obtained energetics can occasionally be rather functional dependent, and we investigate this further below.

Before discussing the spin-states in the two reactions, we note that **2** can form two isomers related through a rotation of the Cu–OH bond, but the energy difference between the two isomers is small (ΔE_{QMMM} = 7 kJ/mol). We here employ the isomer that is lowest in energy, although the small energy difference between them indicate that both most likely will be present in solution.

For the reaction **1** → **2**, we show selected points on the potential energy surface (PES) around the transition state in Figure S3 (TPSS) and Figure S4 (B3LYP), respectively. Before reaching the TS from **1** (left side of Figures S3 and S4), the closed-shell singlet is always higher in energy, but

around the transition state the closed- and open-shell singlet becomes close to identical in energy (for both TPSS and B3LYP). Yet, in the product (**2**), the closed-shell singlet is again higher in energy than the open-shell analog (see Table 1 of the main paper), showing that calculation of spin state energetics indeed is a delicate task.

Along the reaction path, both functionals predict that the singlet and triplet gap will decrease around the transition state. However, for the TPSS functional the decrease is faster. At the TS1, the structures for the singlet and triplet are almost identical, cf. Figure S2 (B), but TPSS predicts a splitting of –29 kJ/mol, i.e., the singlet is 29 kJ/mol *more* stable than the triplet. Thus, using the singlet PES to estimate the activation energy results in a lowering of 29 kJ/mol (from 53 to 24 kJ/mol). Meanwhile, the B3LYP also predicts that the singlet-triplet gap decreases along the reaction path, but for B3LYP the singlet-triplet crossover occurs after TS1 (cf. Figure S3), and B3LYP predicts that the singlet is about 13 kJ/mol *above* the triplet at TS1 cf. Figure S1 (B). As mentioned in the main paper, we have therefore kept the most conservative value (obtained for the triplet PES).

Table S1 contains an analysis of the energy contributions for reaction **1** → **2** due to the protein, i.e., ΔE_{MM} as well as the protein electrostatics, ΔE_{ptch} (cf. equations 1 and 2 in the SI). It can be noted the activation and reaction energy has low contribution from ΔE_{MM} (around 0.5–4 kJ/mol), while the electrostatic contribution (cf. ΔE_{ptch} in Table S5) is significant, highlighting the importance of an QM/MM description: for the activation energy, the electrostatic contributions are between –48 and –29 kJ/mol, depending on functional, while we for the total reaction energy see contributions between of 28 kJ/mol (TPSS) and 14 kJ/mol (B3LYP).

For the reaction **2** → **3**, the closed-shell singlet is higher in energy than the open-shell variant, until reaching the transition state, where the gap is significantly reduced (for TPSS they are essentially degenerate, see Figure S5 and Figure S6). As discussed above, the triplet state is only slightly below the open-shell singlet state in **2** and this is still true around TS2: here the triplet is 11–10 kJ/mol lower, depending on the functional, cf. Figure S2 (E). We have therefore employed the triplet state in calculations of the activation energy (see Figure 3F in the main paper). As seen from Figures S5 and S6, the singlet PES crosses the triplet after TS2, and only the closed-shell form is relevant for **3** (for both functionals). The functionals thus qualitatively agree. However, as also emphasized in the main paper, activation and reaction energies for reaction **2** → **3** are predicted somewhat different by TPSS and B3LYP (see Figure 3F and accompanying text).

The contributions from the environment are also large for reaction **2** → **3** (see Table S2): the MM

contributions are 13 kJ/mol for the activation energy and 3 kJ/mol for the reaction energy, while the electrostatic contributions (ΔE_{ptch}) are between 18 and 25 kJ/mol for the activation energy and –16 and –30 kJ/mol (depending on the functional) for the reaction energy. This again emphasize that (electrostatic) environment contributions are non-negligible.

Table S1: Reaction and activation energies for **1** → **2** (in kJ/mol) All calculations are based on single point calculations with def2-TZVPP basis set on structures optimized with def2-SV(P). O–H is the distance along the reaction coordinate.

Reaction **1→2**	TPSS-D3		B3LYP-D3	
	Transition state	Product	Transition state	Product
$\Delta E_{QM/MM}$	53.3	–69.8	63.9	–48.1
$\Delta E_{QM+ptch}$	57.4	–69.4	67.9	–47.6
ΔE_{MM}	–4.05	–0.4	–4.05	–0.4
ΔE_{QM}	86.3	–41.6	115.6	–33.5
ΔE_{ptch}	–28.9	–27.8	–47.6	–14.2
O–H (Å)	3.62	1.02	3.62	1.02

Table S2: Reaction and activation energies for **2** → **3** (in kJ/mol). All calculations are based on single point calculations with def2-TZVPP basis set on structures optimized with def2-SV(P). O–H is the distance along the reaction coordinate.

Reaction **2→3**	TPSS-D3		B3LYP-D3	
	Transition state	Product	Transition state	Product
$\Delta E_{QM/MM}$	46.1	8.9	78.6	46.7
$\Delta E_{QM+ptch}$	58.5	6.3	91.0	44.3
ΔE_{MM}	–12.4	2.5	–12.4	2.5
ΔE_{QM}	40.1	22.2	66.5	74.2
ΔE_{ptch}	18.4	–15.8	24.5	–30.0
O–H (Å)	1.20	1.00	1.20	1.00

Table S3: Reaction and activation energies for $\mathbf{2}_{RH}$ → **4** (in kJ/mol) All calculations are based on single point calculations with def2-TZVPP basis set on structures optimized with def2-SV(P). C–H is the distance along the reaction coordinate.

Reaction $\mathbf{2}_{RH}$ → **4**	TPSS-D3		B3LYP-D3	
	Transition state	Product	Transition state	Product
$\Delta E_{QM/MM}$	99.2	60.0	108.2	49.7
$\Delta E_{QM+ptch}$	83.6	43.5	92.6	33.4
ΔE_{MM}	6.9	16.4	6.9	16.4
ΔE_{QM}	122.4	89.4	139.1	87.0
ΔE_{ptch}	- 16.0	–45.9	–19.8	–53.6
C–H (Å)	1.60	1.10	1.60	1.10

Table S4: Energies ($E_{QM+ptch}$) in Hartree and $<S^2>$ values for intermediate **1**.

^{3}**1**	TPSS-D3/Def2-TZVPP	B3LYP-D3/Def2-TZVPP
$E_{QM+ptch}$	-3681.580645	-3680.025828
$<S^2>$	2.00595	2.00749
^{1}**1** (open-shell)	TPSS-D3/Def2-TZVPP	B3LYP-D3/Def2-TZVPP
$E_{QM+ptch}$	-3681.57899	-3680.019901
$<S^2>$	0.72810	0.95295

Table S5: Energies ($E_{QM+ptch}$) in Hartree and $<S^2>$ values for intermediate **TS1**.

^{3}**TS1**	TPSS-D3/Def2-TZVPP	B3LYP-D3/Def2-TZVPP
$E_{QM+ptch}$	-3681.558798	-3679.999949
$<S^2>$	2.00565	2.00729
^{1}**TS1** (open-shell)	TPSS-D3/Def2-TZVPP	B3LYP-D3/Def2-TZVPP
$E_{QM+ptch}$	-3681.569688	-3679.994925
$<S^2>$	0.03448	0.48777

Table S6: Energies ($E_{QM+ptch}$) in Hartree and $\langle S^2 \rangle$ values for intermediate **2**.

^{3}**2**	TPSS-D3/De2-TZVPP	B3LYP-D3/De2-TZVPP
$E_{QM+ptch}$	-3681.607084	-3680.04397
$\langle S^2 \rangle$	2.01299	2.03013
^{1}**2** (open-shell)	TPSS-D3/De2-TZVPP	B3LYP-D3/De2-TZVPP
$E_{QM+ptch}$	-3681.605128	-3680.040925
$\langle S^2 \rangle$	0.74743	0.99872

Table S7: Energies ($E_{QM+ptch}$) in Hartree and $\langle S^2 \rangle$ values for intermediate **TS2**.

^{3}**TS2**	TPSS-D3/De2-TZVPP	B3LYP-D3/De2-TZVPP
$E_{QM+ptch}$	-3681.58479	-3680.009313
$\langle S^2 \rangle$	2.00679	2.01629
^{1}**TS2** (open shell)	TPSS-D3/De2-TZVPP	B3LYP-D3/De2-TZVPP
$E_{QM+ptch}$	-3681.58279	-3680.007667
$\langle S^2 \rangle$	0.61913	0.78541

Table S8: Energies ($E_{QM+ptch}$) in Hartree and $\langle S^2 \rangle$ values for intermediate $\mathbf{1}_{RH}$.

$^{3}\mathbf{1}_{RH}$	TPSS-D3/De2-TZVPP	B3LYP-D3/De2-TZVPP
$E_{QM+ptch}$	-4980.421542	-4978.064497
$\langle S^2 \rangle$	2.00664	2.00898
$^{1}\mathbf{1}_{RH}$	TPSS-D3/De2-TZVPP	B3LYP-D3/De2-TZVPP
$E_{QM+ptch}$	-4980.418377	-4978.05766
$\langle S^2 \rangle$	0.72163	0.972010

Table S9: Energies ($E_{QM+ptch}$) in Hartree and $<S^2>$ values for intermediate $\mathbf{2}_{RH}$.

$^{3}\mathbf{2}_{RH}$	TPSS-D3/De2-TZVPP	B3LYP-D3/De2-TZVPP
$E_{QM+ptch}$	-4980.450297	-4978.085524
$<S^2>$	2.01379	2.03063
$^{1}\mathbf{2}_{RH}$	TPSS-D3/De2-TZVPP	B3LYP-D3/De2-TZVPP
$E_{QM+ptch}$	-4980.448500	-4978.085184
$<S^2>$	0.88799	1.0234

Table S10: Reaction energies (in kJ/mol) for $\mathbf{1}_{RH} \rightarrow \mathbf{2}_{RH}$. All calculations are based on single point calculations with def2-TZVPP basis set on structures optimized with def2-SV(P).

Reaction $\mathbf{2}_{RH} \rightarrow \mathbf{4}$	TPSS-D3	B3LYP-D3
$\Delta E_{QM/MM}$	–72.7	–52.4
$\Delta E_{QM+ptch}$	–75.5	–55.2
ΔE_{MM}	2.8	2.8
ΔE_{QM}	–37.9	–22.9
ΔE_{ptch}	–37.5	–32.4

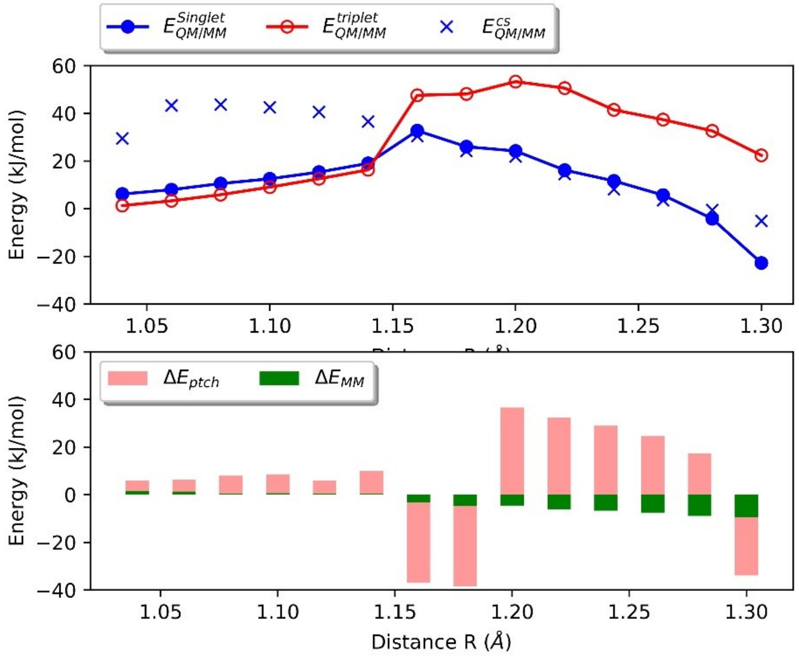

*Figure S3: Selected points near the transition state on the PES of H-transfer from the tyrosine OH group (Tyr164) to the oxyl of the $[CuO]^+$ moiety (**1** → **2**). Calculations are with TPSS-D3/def2-TZVPP employing TPSS-D3/def2-SV(P) structures. The bar plot shows ΔE_{MM} and ΔE_{ptch} for each point.*

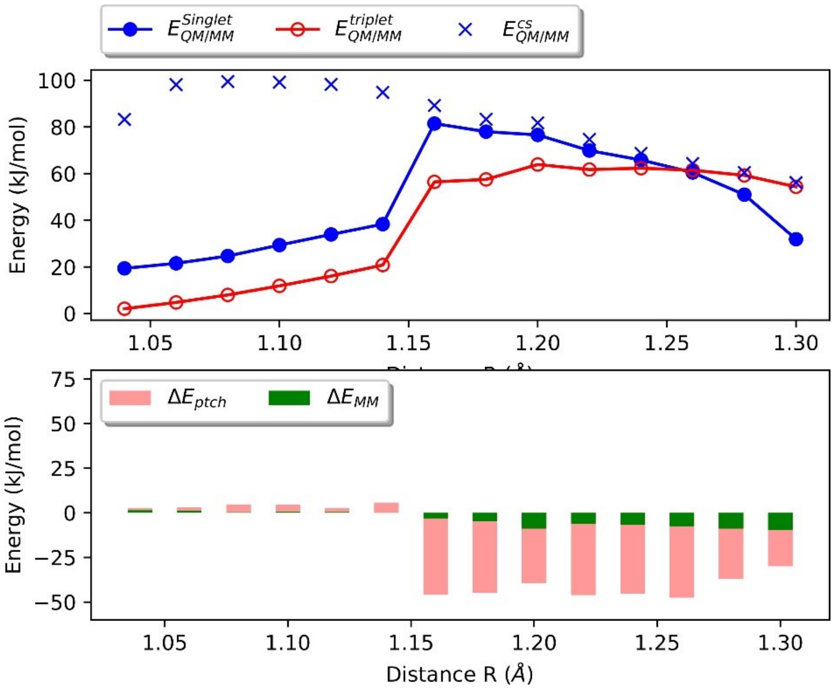

*Figure S4: Selected points along the reaction coordinate (around the transition state) for H-transfer from the tyrosine OH group (Tyr164) to the oxyl of the $[CuO]^+$ moiety (**1** → **2**). Calculations are with B3LYP-D3/def2-TZVPP employing TPSS/def2-SV(P) structures. The bar plot shows ΔE_{MM} and ΔE_{ptch} for each point.*

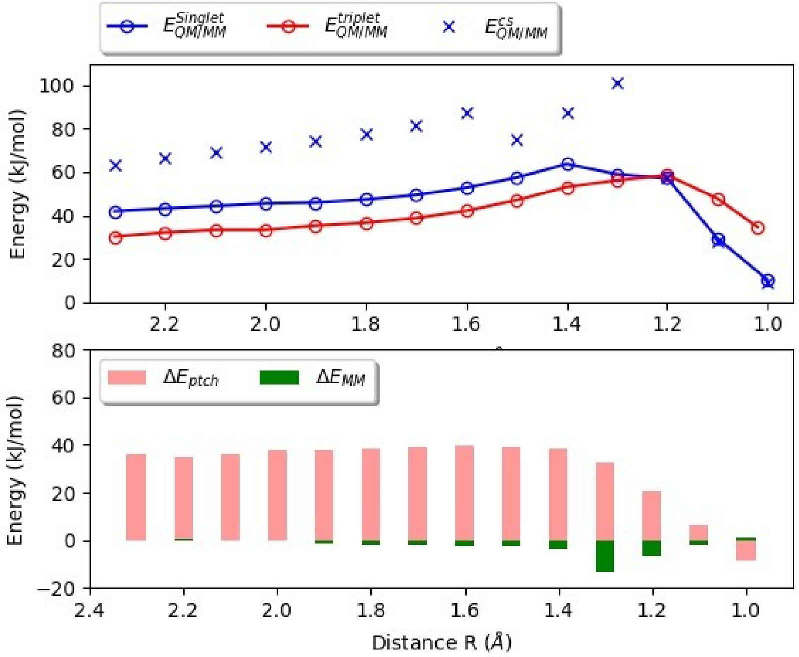

*Figure S5: Selected points along the reaction coordinate (around the transition state) for H-transfer from $W_{ax.}$ to the $[CuOH]^+$ moiety forming trans-$[CuOH]^+$ (**2** → **3**). Calculations are with TPSS-D3/def2-TZVPP employing TPSS-D3/def2-SV(P) structures. The bar plot shows ΔE_{MM} and ΔE_{ptch} for each point.*

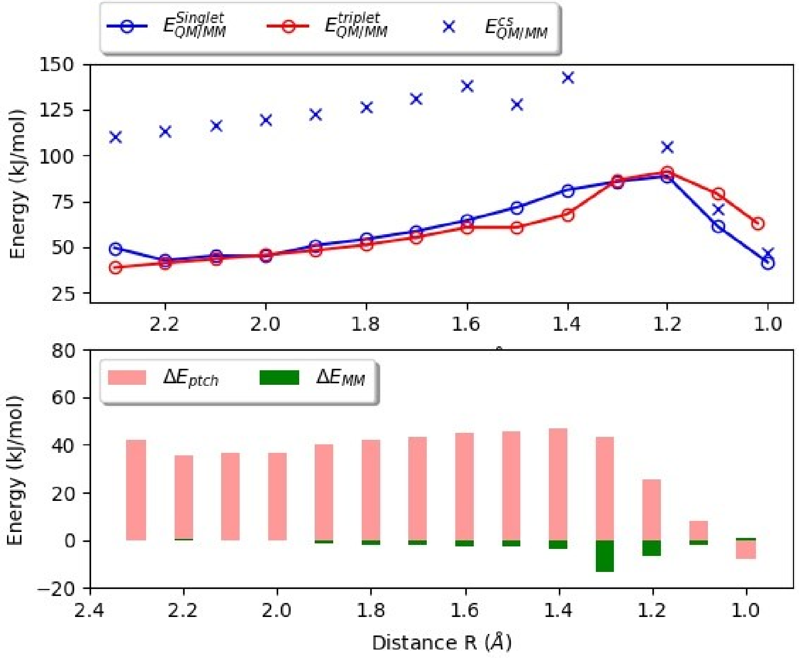

*Figure S6: Selected points along the reaction coordinate (around the transition state) for H-transfer from $W_{ax.}$ to the $[CuOH]^+$ moiety forming trans-$[CuOH]^+$ (**2** → **3**). Calculations are with B3LYP-D3/def2-TZVPP employing TPSS-D3/def2-SV(P) structures. The bar plot shows ΔE_{MM} and ΔE_{ptch} for each point.*

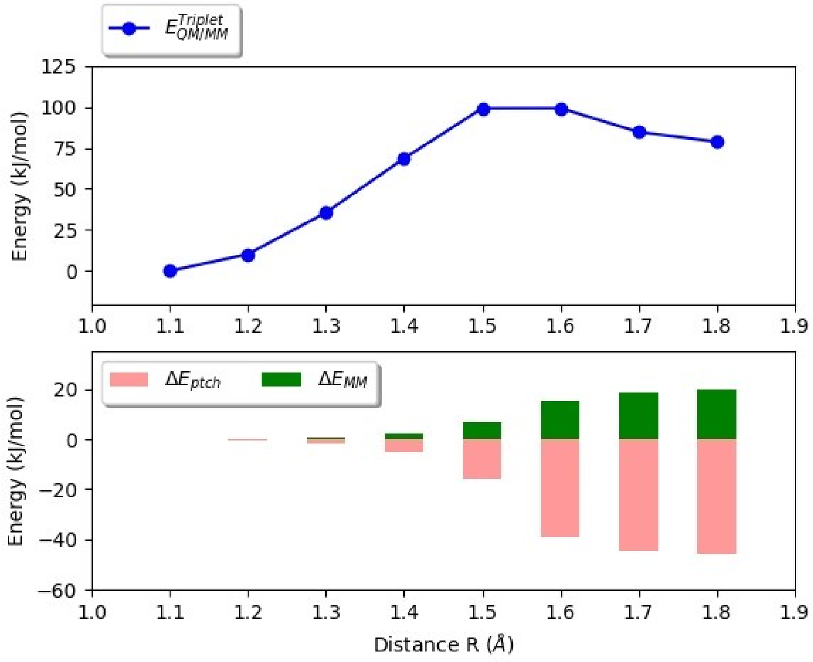

Figure S7: Selected points along the reaction coordinate (around the transition state) for H-transfer from Substrate to the $[CuOH]^{+}$ moiety in $\mathbf{2}_{RH}$ ($\mathbf{2}_{RH} \rightarrow \mathbf{4}$). Calculations are with TPSS-D3/def2-TZVPP employing TPSS-D3/def2-SV(P) structures. The bar plot shows ΔE_{MM} and ΔE_{ptch} for each point.

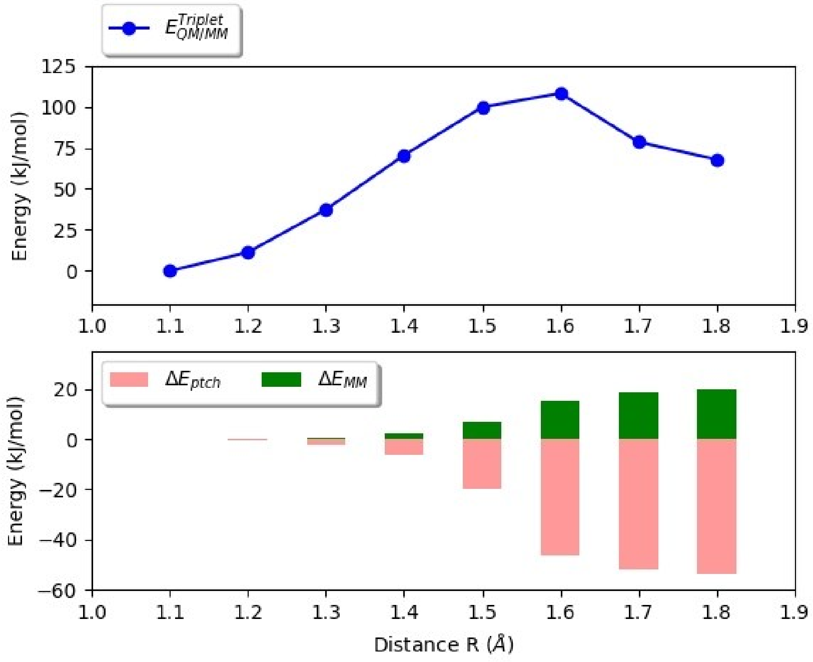

Figure S8: Selected points along the reaction coordinate (around the transition state) for H-transfer from Substrate to the $[CuOH]^{+}$ moiety in $\mathbf{2}_{RH}$ ($\mathbf{2}_{RH} \rightarrow \mathbf{4}$). Calculations are with TPSS-D3/def2-TZVPP employing TPSS-D3/def2-SV(P) structures. The bar plot shows ΔE_{MM} and ΔE_{ptch} for each point.

UV-vis spectra transitions

Table S11. Selected transitions for intermediate **2** (triplet spin-state), calculated with TDDFT, employing CAM-B3LYP/def2-TZVPP.

Label	Δ*E* / nm (eV)	*f*	Orbitals (coefficient)	Assignment
**a**	412 (3.00)	0.039	152B → 161B (0.49)<br>157B → 161B (0.43)<br>158B → 161B (0.43)	$d_{Cu}/n_{Hyd} \rightarrow L_{Tyr}$<br>$L_{His147}/L_{Gln} \rightarrow L_{Tyr}$<br>$L_{His147}/L_{Gln} \rightarrow L_{Tyr}$
**b**	433 (2.85)	0.083	152B → 161B (0.57)<br>158B → 161B (–0.43)	$d_{Cu}/n_{Hyd} \rightarrow L_{Tyr}$<br>$L_{His147}/L_{Gln} \rightarrow L_{Tyr}$
	454 (2.72)	0.001	157B → 161B (0.61)<br>158B → 161B (–0.49)	$L_{His147}/L_{Gln} \rightarrow L_{Tyr}$<br>$L_{His147}/L_{Gln} \rightarrow L_{Tyr}$
**c**	515 (2.40)	0.002	149B → 161B (0.82)	$L_{Tyr} \rightarrow L_{Tyr}$
**c**	540 (2.29)	0.004	159B → 161B (0.86)	$L_{Gln} \rightarrow L_{Tyr}$
**c**	542 (2.28)	0.0001	132B → 162B (–0.56)	$d_{Cu} \rightarrow d_{Cu}$

Table S12. Selected transitions for intermediate **2** (open-shell singlet), calculated with TDDFT, employing CAM-B3LYP/def2-TZVPP.

Label	Δ*E* / nm (eV)	*f*	Orbitals (coefficient)	Assignment
**a**	388.0 (3.20)	0.098	146A → 162A (–0.38)<br>148A → 162A (0.37)<br>149A → 162A (0.44)<br>153A → 162A (0.46)	$d_{Cu}/n_{Hyd} \rightarrow L_{Tyr}$<br>$L_{Gln}/ L_{His147} \rightarrow L_{Tyr}$<br>$d_{Cu} \rightarrow L_{Tyr}$<br>$n_{Hyd} \rightarrow L_{Tyr}$
**b**	426.1(2.91)	0.011	155A → 162A (–0.43)<br>159A → 162A (0.75)	$L_{Gln} \rightarrow L_{Tyr}$<br>$L_{His147} \rightarrow L_{Tyr}$
	438.9 (2.83)	0.001	157A → 162A (0.66)<br>158A → 162A (–0.51)	$n_{Hyd} \rightarrow L_{Tyr}$<br>$L_{Gln}/L_{His147} \rightarrow L_{Tyr}$
	495.9 (2.50)	0.000	156A → 162A (0.63)<br>157A → 162A (–0.43)<br>158A → 162A (–0.37)	$L_{Gln}/L_{His147} \rightarrow L_{Tyr}$<br>$n_{Hyd} \rightarrow L_{Tyr}$<br>$L_{Gln}/L_{His147} \rightarrow L_{Tyr}$
	502.1 (2.47)	0.001	156A → 162A (–0.31)<br>133B → 162B (–0.43)<br>138B → 162B (–0.28)<br>141B → 162B (0.41)<br>146B → 162B (–0.28)	$L_{Gln}/L_{His147} \rightarrow L_{Tyr}$<br>$d_{Cu} \rightarrow d_{Cu}$<br>$d_{Cu} \rightarrow d_{Cu}$<br>$d_{Cu} \rightarrow d_{Cu}$<br>$d_{Cu} \rightarrow d_{Cu}$
	516.7 (2.40)	0.002	150A → 162A (0.77)<br>160A → 162A (0.44)	$L_{Tyr} \rightarrow L_{Tyr}$<br>$L_{Gln} \rightarrow L_{Tyr}$
	522.2 (2.37)	0.000	135B → 162B (–0.45)<br>140B → 162B (–0.37)<br>146B → 162B (–0.33)<br>147B → 162B (–0.42)	$d_{Cu} \rightarrow d_{Cu}$<br>$d_{Cu} \rightarrow d_{Cu}$<br>$d_{Cu} \rightarrow d_{Cu}$<br>$d_{Cu} \rightarrow d_{Cu}$
**c**	535.0 (2.32)	0.007	150A → 162A (–0.48)<br>160A → 162A (0.74)	$L_{Tyr} \rightarrow L_{Tyr}$<br>$L_{Gln} \rightarrow L_{Tyr}$

Table S13. Selected transitions for intermediate **3** (closed-shell spin state) calculated with TDDFT, employing CAM-B3LYP/def2-TZVPP.

Label	Δ*E* / nm (eV)	*f*	Orbitals (coefficient)	Assignment
	406.7 (3.05)	0.002	152 → 162 (0.46)<br>155 → 162 (0.31)	$d_{Cu} \rightarrow d_{Cu}$
	421.6 (2.94)	0.006	154 → 162 (0.61)	$L_{Tyr} \rightarrow d_{Cu}$
	425.5 (2.91)	0.001	131 → 162 (0.40)<br>146 → 162 (0.28)<br>154 → 162 (0.27)	$d_{Cu} \rightarrow d_{Cu}$<br>$L_{His78} \rightarrow d_{Cu}$<br>$L_{Tyr} \rightarrow d_{Cu}$
	437.7 (2.83)	0.001	156 → 162 (0.60)	$L_{Gln} \rightarrow d_{Cu}$
**a**	530.3 (2.34)	0.207	148 → 162 (0.36)<br>160 → 162 (0.45)	$n_{Hyd} \rightarrow d_{Cu}$<br>$L_{Tyr} \rightarrow d_{Cu}$
**b**	577.4 (2.15)	0.060	157 → 162 (–0.32)<br>159 → 162 (0.45)	$L_{His147} \rightarrow d_{Cu}$<br>$L_{His147} \rightarrow d_{Cu}$
**d**	635.9 (1.95)	0.035	148 → 162 (0.47)	$n_{Hyd} \rightarrow d_{Cu}$
**d**	762.2 (1.63)	0.017	151 → 162 (0.30)<br>161 → 162 (0.45)	$n_{Tyr} \rightarrow d_{Cu}$<br>$L_{His147} \rightarrow d_{Cu}$
**d**	767.6 (1.62)	0.011	151 → 162 (0.55)	$n_{Tyr} \rightarrow d_{Cu}$
**e**	975.7 (1.27)	0.021	149 → 162 (– 0.36)<br>155 → 162 (0.39)<br>160 → 162 (0.33)	$L_{His1} \rightarrow d_{Cu}$<br>$L_{His1} \rightarrow d_{Cu}$<br>$L_{Tyr} \rightarrow d_{Cu}$

Table S14. Selected transitions for intermediate **3** (triplet spin state) calculated with TDDFT, employing CAM-B3LYP/def2-TZVPP.

Label	Δ*E* / nm (eV)	*f*	Orbitals (coefficient)	Assignment
	366.54 (3.38)	0.013	144B → 161B (–0.47)<br>151B → 161B (0.53)	L_{Gln} → L_{Tyr}<br>L_{His1} → L_{Tyr}
	389.74 (3.18)	0.001	156B → 161B (0.90)	L_{His147} → L_{Tyr}
	394.78 (3.14)	0.002	157B → 161B (0.88)	L_{His147} / L_{Gln} → L_{Tyr}
	396.98 (3.12)	0.008	153B → 161B (0.85)<br>157B → 161B (0.43)	n_{Hyd} → L_{Tyr}<br>L_{Gln} → L_{Tyr}
**a**	408.20 (3.04)	0.075	150B → 161B (0.43)<br>152B → 161B (0.52)	n_{Tyr} → L_{Tyr}<br>d_{Cu} / n_{Hyd} → L_{Tyr}
	441.90 (2.81)	0.001	154B → 161B (0.87)	n_{Hyd} → L_{Tyr}
	467.74 (2.65)	0.000	155B → 161B (0.78)	L_{His147} → L_{Tyr}
	558.45 (2.22)	0.004	148B → 161B (0.77)<br>149B → 161B (0.55)	L_{His1} → L_{Tyr}<br>L_{Tyr} → L_{Tyr}
	561.50 (2.21)	0.001	158B → 161B (0.68)<br>159B → 161B (–0.56)	L_{Gln} → L_{Tyr}<br>L_{Gln} → L_{Tyr}
	605.43 (2.04)	0.001	137B → 162B (0.54)	L_{Gln} → d_{Cu}
	625.54 (1.98)	0.002	132B → 162B (0.36)<br>133B → 162B (0.37)<br>137B → 162B (– 0.39)<br>146B →162B (–0.40)	d_{Cu} → d_{Cu}<br>L_{Gln} → d_{Cu}<br>L_{Gln} → d_{Cu}<br>d_{Cu} / L_{His78} → d_{Cu}
**b**	689.23 (1.80)	0.053	158B → 161B (0.50)<br>159B → 161B (0.65)	L_{Gln} → L_{Tyr}<br>L_{Gln} → L_{Tyr}
	725.05 (1.71)	0.000	140B → 162B (–0.41)<br>144B → 162B (0.54)<br>155B → 162B (0.44)	d_{Cu} → d_{Cu}<br>L_{Gln} → d_{Cu}<br>L_{His147} → d_{Cu}
	774.14 (1.60)	0.000	160B → 161B (1.00)	L_{His147} → L_{Tyr}
	965.84 (1.28)	0.003	146B →161B (0.30)	L_{His78} → L_{Tyr}

			147B → 161B (0.65)<br>150B → 161B (–0.40)	$L_{His1} \rightarrow L_{Tyr}$<br>$n_{Tyr} \rightarrow L_{Tyr}$

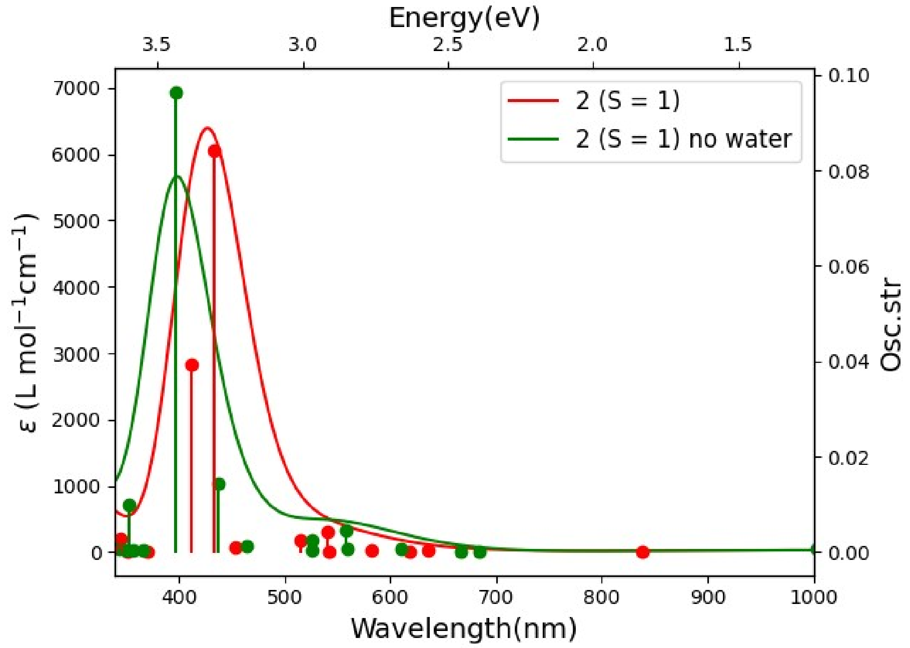

*Figure S9: Spectra for **2** (triplet) calculated with and without axial water (W_{ax}). The spectra are calculated with CAM-B3LYP/def2-TZVPP on (truncated) TPSS-D3/def2-SV(P) QM/MM structures.*

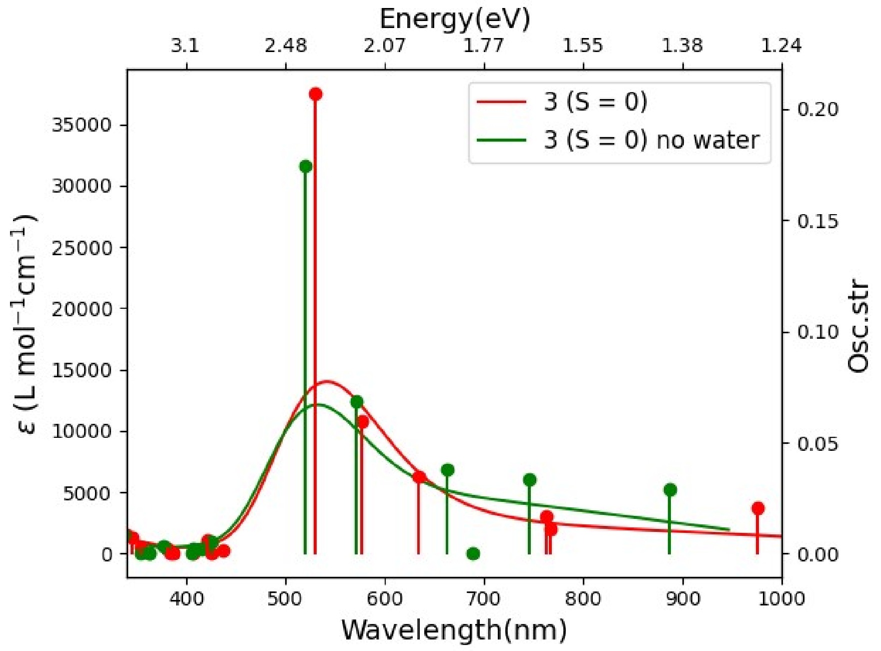

*Figure S10: Spectra for **3** (singlet) calculated with and without equatorial water (W_{eq}). The spectra are calculated with CAM-B3LYP/def2-TZVPP on (truncated) TPSS-D3/def2-SV(P) QM/MM structures.*

References

1. Hedegård, E. D. & Ryde, U. *Chem. Sci.* **9,** 3866–3880 (2018).
2. Frandsen, K. E. H., Simmons, T. J., Dupree, P., Poulsen, J.-C. N., Hemsworth, G. R., Ciano, L., Johnston, E. M., Tovborg, M., Johansen, K. S., Freiesleben, P. Von, Marmuse, L., Fort, S., Cottaz, S., Driguez, H., Henrissat, B., Lenfant, N., Tuna, F., Baldansuren, A., Davies, G. J., *et al. Nat. Chem. Biol.* **12,** 298–305 (2016).
3. Singh, R. K., Blossom, B. M., Russo, D. A., Singh, R., Weihe, H., Andersen, N. H., Tiwari, M. K., Jensen, P. E., Felby, C., & Bjerrum, M. J. *Chem. – A Eur. J.* **26,** 454 –463 (2020).
4. Paradisi, A., Johnston, E. M., Tovborg, M., Nicoll, C. R., Ciano, L., Dowle, A., Mcmaster, J., Hancock, Y., Davies, G. J., & Walton, P. H. *J. Am. Chem. Soc.* **141,** 18585–18599 (2019).
5. Hedegård, E. D. & Ryde, U. *ACS Omega* **2,** 536–545 (2017).
6. Ryde, U. *J. Comput. Aided. Mol. Des.* **10,** 153–164 (1996).
7. Reuter, N., Dejaegere, A., Maigret, B., & Karplus, M. *J. Phys. Chem. A* **104,** 1720–1735 (2000).
8. Hu, L. & Ryde, U. *J. Chem. Theory Comput.* **7,** 2452–2463 (2011).
9. Larsson, E. D., Dong, G., Veryazov, V., Ryde, U., & Hedegård, E. D. *Dalt. Trans.* **49,** 1501–1512 (2020).
